# Genetic and clinical characteristics of pediatric patients with cystic fibrosis: a single-center retrospective study in China

**DOI:** 10.1186/s13023-026-04290-w

**Published:** 2026-03-10

**Authors:** Jinrong Liu, Lanhong Ma, Weijian Yu, Jin Zhou, Guanghua Zhou, Yilijang Tuhongjiang, Gulimire Maitusong, Ziwei Wang, Shapaguli Aizeze, Dianhui Tian, Xiaoyan Zhang, Haiming Yang

**Affiliations:** 1 Department II of Respiratory Diseases, National Clinical Research Center for Respiratory Diseases, Beijing Children’s Hospital, Capital Medical University, National Center for Children’s Health, Beijing, China; 2 Department of Respiratory Diseases, Children’s Hospital of Xinjiang Uygur Autonomous Region, Xinjiang Hospital of Beijing Children’s Hospital, Pediatric Research Institute of Xinjiang Uygur Autonomous Region,, The Seventh People’s Hospital of Xinjiang Uygur Autonomous Region, No. 393, Altay Road, Saibak District, Urumqi, 830000 China; 3 Department of Gastroenterology, Beijing Children’s Hospital, Capital Medical University, National Center for Children’s Health, Beijing, China

**Keywords:** Cystic fibrosis, Genotype, Phenotype, China

## Abstract

**Background:**

Despite the increasing recognition of cystic fibrosis (CF) in China, few cases have been reported in the Xinjiang Uyghur Autonomous Region (Xinjiang), which is located in China’s northwest with a diverse ethnic composition. This study aimed to describe the genotype and clinical phenotype of children with CF in Xinjiang.

**Methods:**

We recruited children diagnosed with CF at the Children’s Hospital of Xinjiang Uygur Autonomous Region between January 2012 and December 2025. The demographic data, imaging findings, laboratory test results, and genetic data were retrospectively reviewed.

**Results:**

A total of 19 patients from 18 families were enrolled. The median age at diagnosis was 9.8 years. Among them, 15 (78.9%) were Uygur, 3 (15.8%) Kazakh, and 1 (5.3%) Mongolian. Fifteen distinct cystic fibrosis transmembrane conductance regulator (*CFTR)* mutations were identified, with c.1521_1523delCTT (p. F508del) being the most common (allele frequency: 36.1%). Mutations of c.1860T > G (p.H620Q), c.2991 G > C  (p.L997F), del ex4-11, c.3254 A > G (p.H1085R), c.2619 + 1G > A, c.349 C > T (p.R117C), c.3909 C > G (p.N1303K), and c.1911delA > G (p.G637Hfs*26)—which have never been reported in Chinese populations—were observed. Bronchiectasis was observed in 84.2% of patients, with allergic bronchopulmonary aspergillosis noted in 36.8%. *Pseudomonas aeruginosa* (78.9%) and *Staphylococcus aureus* (47.4%) were the predominant pathogens. Additionally, 78.9% had pancreatic insufficiency, 21.1% had CF-related liver disease, 21.1% had Pseudo-Bartter syndrome, and 5.3% had diabetes.

**Conclusion:**

CF may be significantly underdiagnosed in Xinjiang, China. The genotypic spectrum in children with CF in this multiethnic region differs considerably from previous reports, mainly focusing on the Han population, with p.F508del as the most frequent *CFTR* mutation.

## Introduction

Cystic fibrosis (CF) is a life-limiting autosomal recessive disorder caused by biallelic pathogenic mutations in the cystic fibrosis transmembrane conductance regulator *(CFTR)* gene. Although CF is a monogenic disease, its incidence and the diversity of *CFTR* gene variants exhibit notable racial and regional disparities [1]. CF is mostly reported in the Caucasian population, with an incidence of 1/25,000 ~ 1/1800, while the reported CF prevalence in East Asian countries is much lower [2].

China, a vast multiethnic nation, comprises 56 distinct ethnic groups. The Han people constitute approximately 90% of the total population. To date, only about 200 CF cases of Chinese origin have been reported [3]. These studies indicate the *CFTR* mutation spectrum in the Chinese population differs distinctly from that in Caucasians [4–6]. Reported cases are predominantly concentrated among the Han people in northern, eastern, central, and southern China [6, 7]. However, epidemiological data from the Xinjiang Uygur Autonomous Region (Xinjiang), China’s largest provincial-level administrative division and a northwest border region with numerous minority ethnic groups, remain notably lacking. Additionally, Xinjiang is also the heart of the ancient Silk Road and has historically experienced migration from many Eurasian groups [8, 9]. Despite this unique demographic context, the genotypic and clinical features of pediatric CF cases in Xinjiang have not been systematically studied.

In this study, we retrospectively analyzed the clinical data and *CFTR* mutations in 19 children with CF from a single tertiary pediatric respiratory ward in Xinjiang. Our findings provide new insights into CF among the Chinese population and a deepening understanding of the *CFTR* spectrum, which will significantly contribute to the future screening and treatment strategies for this disease in China.

## Methods

A retrospective analysis was conducted on 19 Chinese children with CF hospitalized in the Department of Respiratory Diseases at Children’s Hospital of Xinjiang Uygur Autonomous Region from January 2012 to December 2025. CF was diagnosed based on the Consensus Guidelines from the Cystic Fibrosis Foundation 2017: at least one of the clinical features highly suggestive of CF and evidence of *CFTR* dysfunction, including elevated sweat test and/or presence of biallelic pathogenic variants [10]. The basic information, clinical manifestations, laboratory test results, chest imaging findings, and gene mutations at diagnosis were analyzed retrospectively through the hospital’s electronic medical record system. Measurement data were given as rates and percentages, and enumeration data as medians (interquartile ranges, IQR). Mutation screening of *CFTR*: Genomic DNA samples were extracted from peripheral blood leukocytes using standard genomic DNA purification methods. Whole-exome sequencing, bioinformatics analysis, and Sanger sequencing validation were performed according to the standard approach as previously described [11]. Multiplex ligation-dependent probe amplification (MLPA, MRC-Holland) was applied to detect the large deletions or duplications in the *CFTR* gene. All exons, proximal introns, and selected regions of deep introns were thoroughly analyzed. Besides, *CFTR* gene sequencing was performed using Sanger sequencing in one patient (Patient 1) in 2012.

Informed consent was obtained from the legal guardians of all the participants. This study was conducted in accordance with the tenets of the amended Declaration of Helsinki and approved by the ethics committee of the Children’s Hospital of Xinjiang Uygur Autonomous Region (KY2022101709).

## Results

**Fig. 1:**
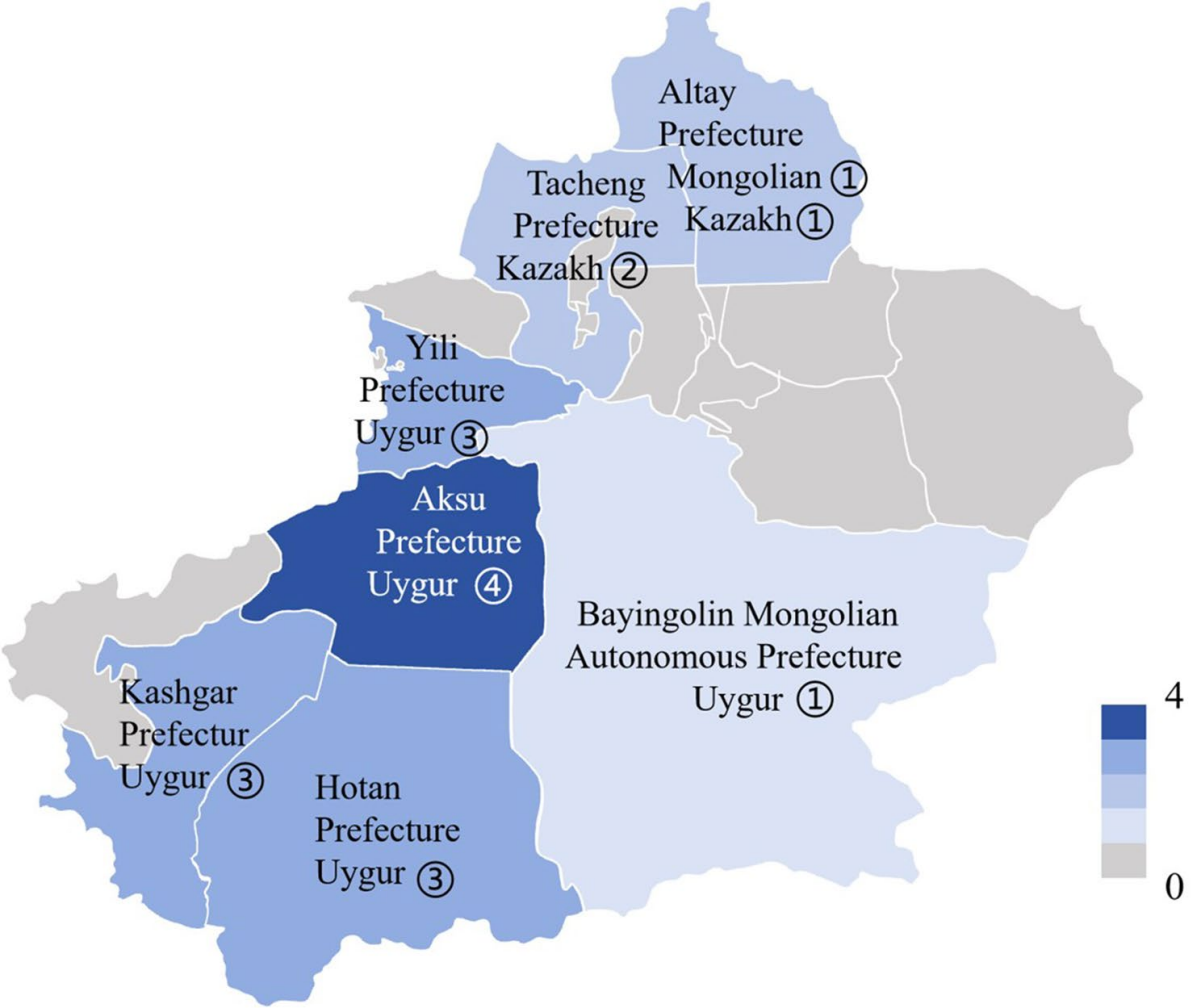
The regional distribution of the 18 CF families in Xinjiang, China. Arabic numerals indicate the number of families in different regions

A total of 19 pediatric CF patients (11 males and 8 females) from 18 Chinese families were recruited in this study. Among them, 17 (89.5%) were diagnosed after January 2022. Additionally, 5 (26.3%) were infants, and 3 (15.8%) of these infants were diagnosed after July 2025. Furthermore, 15 (78.9%) were of Uygur ethnicity, 3 (15.8%) were of Kazakh ethnicity, and 1 (5.3%) was of Mongolian ethnicity. The median age at symptom onset was 0.5 years (IQR: 0.2–3.3 years), while the median age at diagnosis was 9.8 years (IQR: 3.2–14.8 years). The median delay in diagnosis was 6.3 years (IQR: 2.0–11.9 years), calculated from symptom onset to diagnosis. Patients 9–1 and 9–2 were siblings, both suffering from CF. The parents of all the patients denied having consanguineous marriages within their families. The regional distribution of the 18 CF families in Xinjiang is shown in Fig. 1. Although patient 1 has been previously reported [12, 13], she has been admitted to our hospital for treatment on several occasions.

### *CFTR* gene mutations

*CFTR* gene variants were identified in all patients. Among them, 11 patients exhibited homozygous mutations, while 8 displayed compound heterozygous mutations. A total of 15 distinct mutations were detected, including 8 missense mutations, 3 in-frame mutations, 3 splicing mutations, and 1 large deletion. Among the 18 unrelated probands, the c.1521_1523delCTT (p. F508del) variant was the most frequent, with an allele frequency of 36.1% (13/36 alleles). Additionally, mutations such as c.2619 + 1G > A (3/36 alleles, 8.3%), c.1860T > G (p.H620Q) (2/36 alleles, 5.6%), c.2991G> C (p.L997F) (2/36 alleles, 5.6%), del ex4-11 (2/36 alleles, 5.6%), c.3254 A > G (p.H1085R) (1/36 alleles, 2.8%), c.349 C > T (p.R117C) (1/36 alleles, 2.8%), c.3909 C > G (p.N1303K) (1/36 alleles, 2.8%), and c.1911del (p.G637Hfs*26) (1/36 alleles, 2.8%)—which have not been previously reported in Chinese populations—were observed.

The identified *CFTR* variants in the 19 patients (from 18 families) with CF are detailed in Table 1.

**Table 1:** Mutations of *CFTR* identified in the 19 children (from 18 families) with CF from this study

Pts	Region	Nucleotide change	Amino acid change	Mutation type	Genotype	Source
P1	Ex 20	c.3196 C > T	p.Arg1066Cys	Missenses	Homozygous	P/M
P2	Ex 11	c.1521_1523delCTT	p.Phe508del	In-frame	Homozygous	P/M
P3	Ex 11	c.1521_1523delCTT	p.Phe508del	In-frame	Homozygous	P/M
P4	Ex 11	c.1521_1523delCTT	p.Phe508del	In-frame	Homozygous	P/M
P5	Ex 11	c.1521_1523delCTT	p.Phe508del	In-frame	Homozygous	P/M
P6	Ex 11	c.1521_1523delCTT	p.Phe508del	In-frame	Homozygous	P/M
P7	Ex 14	c.1860T > G	p.His620Gln	Missenses	Homozygous	P/M
P8	Ex 20 Ex 25	c.3196 C > T c.4111_4113dup	p.Arg1066Cys p.Glu1371dup	Missenses In-frame	Compound heterozygous	M P
P9-1	In 16 In 22	c.2658-1G > C c.3718–2477 C	- -	Splicing Splicing	Compound heterozygous	M P
P9-2	In 16 In 22	c.2658-1G > C c.3718–2477 C	- -	Splicing Splicing	Compound heterozygous	M P
P10	Ex 4–11	28.98Kb deletion	-	Large deletion	Homozygous	P/M
P11	Ex 13 Ex 18	c.1733T > C c.2909G > A	p.L578P p.G970D	Missenses Missenses	Compound heterozygous	M P
P12	Ex 11 In 5	c.1521_1523delCTT c.2619 + 1G > A	p.Phe508del -	In-frame Splicing	Compound heterozygous	M P
P13	Ex 11 Ex 20	c.1521_1523delCTT c.3196 C > T	p.Phe508del p.Arg1066Cys	In-frame Missenses	Compound heterozygous	M P
P14	Ex 20	c.3254 A > G	p.His1085Arg	Missenses	Homozygous	P/M
P15	Ex 20	c.2991G > C	p.Leu997Phe	Missenses	Homozygous	P/M
P16	Ex 4 Ex 24	c.349 C > T c.3909 C > G	p.Arg117Cys p.Asn1303Ly	Missenses Missenses	Compound heterozygous	M P
P17	Ex 11 Ex 14	c.1521_1523del c.1911del	(p.Phe508del) (p.Gln637HisfsTer26)	In-frame In-frame	Compound heterozygous	M P
P18	In 5	c.2619 + 1G > A	-	Splicing	Homozygous	P/M

Ex: Exon, Pts: Patients, M: Maternal, P: Paternal

### Clinical manifestations

The respiratory system was the most commonly affected, with 73.7% (14/19) of patients being hospitalized due to persistent respiratory symptoms, while 26.3% (5/19) were admitted primarily for gastrointestinal issues. Nutritional assessments, encompassing body mass index (BMI) and percentile rankings, revealed malnutrition in all patients. The median BMI was 13.8 kg/m^2^ (IQR: 12.2–14.9 kg/m^2^), with low weight percentiles; specifically, 78.9% (15/19) ranked at or below the 3rd percentile, and 15.8% (3/19) fell between the 3rd and 10th percentiles.

**Fig. 2:**
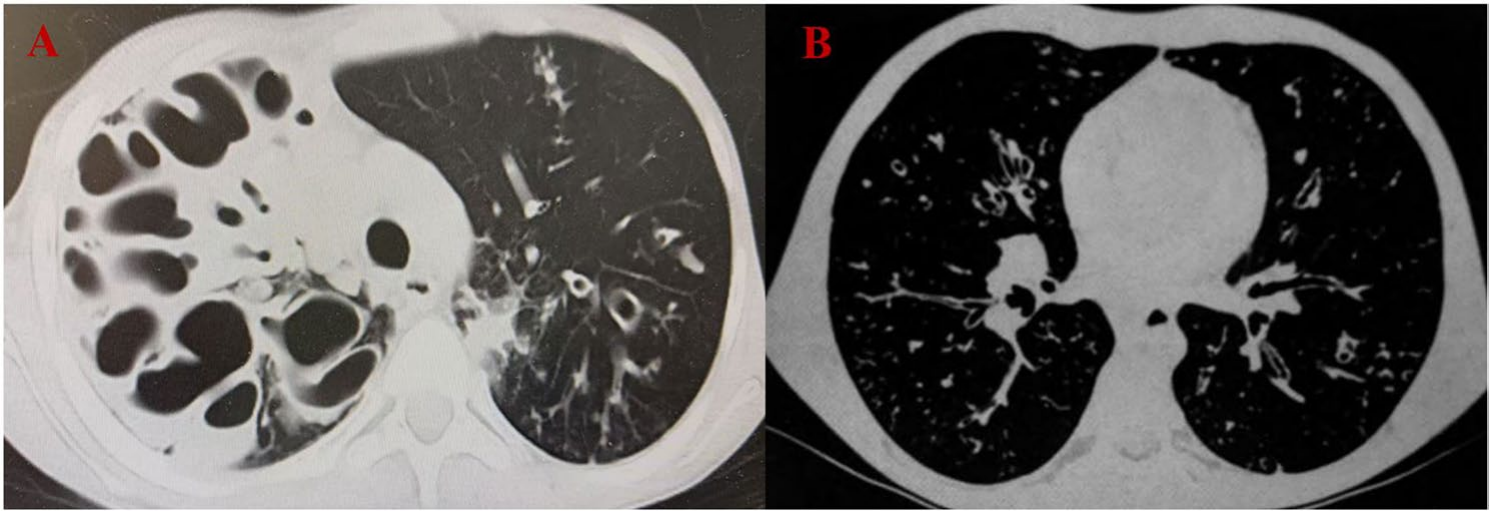
Chest CT images of children diagnosed with CF. (**A**) Chest CT of patient 8 shows extensive bronchial dilation within hyperdense lesions in the right lung; multiple bronchial wall thickening and luminal dilation are identified in the left lung, with some lesions showing the signet ring. (**B**) Chest CT of patient 1 showed multiple bronchial wall thickening and bronchial luminal dilation in both lungs, as well as small centrilobular nodules in the right lung

Most patients (16/19, 84.2%) presented with both chronic productive cough and bronchiectasis. Recurrent or persistent pneumonia was observed in 63.2% (12/19) of patients, chronic sinusitis in 68.4% (13/19), and allergic bronchopulmonary aspergillosis (ABPA) in 36.8% (7/19). Finger clubbing was observed in 63.2% (12/19). Sputum cultures revealed *Pseudomonas aeruginosa (P. aeruginosa)* as the predominant pathogen (15/19, 78.9%), followed by *Staphylococcus aureus (S. aureus)* (9/19, 47.4%). Twelve children underwent pulmonary function testing, revealing a median forced expiratory volume in one second (FEV1) of 59.4% (IQR: 48.7–82.4%); among them, 75.0% (9/12) had FEV1 below 80% of predicted values. Half of these (6/12, 50.0%) also had reduced forced vital capacity [77.4% (IQR: 69.4–86.8%)]. Patient 8 exhibited the most severe lung structural damage on chest computed tomography (CT) scan, as shown in Fig. 2A. Notably, Patient 1 (homozygous c.3196 C > T, p.R1066C mutation) died of cardiopulmonary failure at the age of 11 years (chest CT shown in Fig. 2B).

Gastrointestinal manifestations included pancreatic insufficiency (PI) in 78.9% (15/19) of patients, all presenting with malnutrition and steatorrhea; notably, low fecal elastase (FE) levels were observed in 11 cases (57.9%) (Table 2). Additionally, 21.1% (4/19) had CF-related liver disease (CFLD), characterized by elevated liver enzymes, cholestasis, cirrhosis, and portal hypertension. Diabetes mellitus co-occurred in one patient (5.3%). No cases of pancreatitis were observed. Pseudo-Bartter syndrome was diagnosed in 4 patients (21.0%, 4/19).

**Table 2:** Demographic date and clinical manifestations of the 19 children (from 18 families) with CF from this study

Pts	Ethnic group	Sex	AoO (year)	Age at Dx (year)	Weight Centile, BMI	Respiratory manif	Digestive systems manif	Metabolic systems manif	Sputum pathogens	Pulmonary function FEV1% FVC%	FE-1(ug/g)	Sweat test (mmol/L)
P1	Uygur	F	0.08	10.3	< P3, 13.9	Recurrent productive cough, Bronchiectasis	Suspected PI	None	*PA*, *A. fumigatus*	42.2 72.4	ND	ND
P2	Uygur	M	0.08	0.92	< P3, 11.0	Recurrent productive cough, wheezing, Bronchiectasis ABPA	PI	None	*PA*,* E. coli*	ND	<5	108
P3	Uygur	M	0.50	7.42	P3, 13.6	Recurrent productive cough wheezing, Bronchiectasis, ABPA	CFLD, PI	None	*PA*	117.5 116.9	<5	ND
P4	Kazakh	M	6.00	11.67	P3-P10, 13.7	Recurrent productive cough, Bronchiectasis, ABPA	CFLD, PI	None	*S.aureus*,* PA*,* B.cepacia*	56.4 69.3	<5	129
P5	Kazakh	M	0.25	15.92	< P3, 15.2	Recurrent productive cough, wheezing, Bronchiectasis, ABPA	PI	Diabetes, PBS	*PA*, *H. influenzae*,* A.xylosoxidans*	48.0 69.8	<5	117
P6	Uygur	M	1.00	8.25	< P3, 13.5	Recurrent productive cough, Bronchiectasis,	suspected PI	None	*PA*,* S.aureus*	73.8 81.2	ND	ND
P7	Uygur	F	0.14	14.83	P3-P10, 17.3	Recurrent productive cough, wheezing, Bronchiectasis,	PI	None	*PA*	59.2 73.6	65	ND
P8	Kazakh	M	3.00	13.92	< P3, 12.5	Recurrent productive cough, wheezing, Bronchiectasis, ABPA	PI	None	*PA*,* S.aureus*	59.5 63.3	<5	ND
P9-1	Uygur	M	8.00	10.42	P3, 14.7	Recurrent productive cough, Bronchiectasis	Elevated liver enzymes	None	*S.aureus*,* PA*, *A. fumigatus*	85.3 99.3	ND	ND
P9-2	Uygur	F	6.00	16.50	P3-P10, 16.6	Recurrent productive cough, Bronchiectasis	None	None	-	ND	ND	ND
P10	Uygur	M	3.00	8.67	< P3, 10.8	Recurrent productive cough, wheezing, Bronchiectasis	PI	None	*PA*	86.4 96.9	<5	ND
P11	Uygur	M	1.50	3.92	< P3, 11.3	Recurrent productive cough, Bronchiectasis	CFLD, Suspected PI	None	*PA*, *H. influenzae*	ND	ND	ND
P12	Uygur	M	0.4	0.6	< P3, 13.8	Recurrent productive cough, Bronchiectasis	CFLD	None	*PA*, *S. maltophilia*	ND	ND	ND
P13	Uygur	M	0.3	15.1	< P3, 14.3	Recurrent productive cough, wheezing, Bronchiectasis, ABPA	PI	None	*PA*, *H. influenzae*, *S.aureus*	50.6 85.3	<5	ND
P14	Mongolian	F	4.0	9.25	P25-P50, 15.3	Recurrent productive cough, wheezing, Bronchiectasis	PI	None	*PA*,* S.aureus* *C. albicans*	73.4 86.4	12	ND
P15	Uygur	F	0.08	15.08	< P3, 14.5	Recurrent productive cough, wheezing, ABPA Bronchiectasis	Suspected PI	None	*PA*,* S.aureus*	34.3 43.7	ND	ND
P16	Uygur	F	0.3	0.8	< P3, 14.3	None	None	PBS	*S.aureus*, *S. pneumoniae*	ND	ND	ND
P17	Uygur	F	0.3	0.4	< P3, 10.2	Intermittent cough	PI	PBS	*S.aureus*, *H. influenzae*	ND	39	ND
P18	Uygur	F	0.1	0.3	< P3, 11.9	None	PI	PBS	*M. catarrhalis*	ND	69.9	ND

AoO: Age of symptom onset, ABPA: Allergic bronchopulmonary aspergillosis, *A. xylosoxidans: Achromobacter xylosoxidans*, *A. fumigatus: Aspergillus fumigatus*，BMI: Body mass index, *B. cepacia: Burkholderia cepacia*, CFLD: Cystic ﬁbrosis-related liver disease, *C. albicans: Candida albicans*, Dx: diagnosis, *E. coli: Escherichia coli*, FE-1: Fecal elastase-1, F: Female, FEV1: Forced Expiratory Volume in one second, FVC: Forced Vital Capacity, *H. influenzae: Haemophilus influenzae*, M: Male, Manif: Manifestations, *M. catarrhalis: Moraxella catarrhalis*, ND: Not Done, *PA: Pseudomonas aeruginosa*, PI: Pancreatic insufficiency, Pts: Patients, PBS: Pseudo-Barta syndrome, S. aureus: Staphylococcus aureus, *S. pneumoniae: Streptococcus pneumoniae*, *S. maltophilia: Stenotrophomonas maltophilia*.

Demographic and clinical characteristics of the 19 children with CF (from 18 families) are summarized in Table 2.

## Discussion

To our knowledge, this is the first study to describe the genetic and clinical characteristics of children with CF in Xinjiang, China. Xinjiang, which covers one-sixth of China’s land area, has a diverse ethnic composition, including Uygur (43.6%), Han (40.6%), Kazakh (8.3%), and numerous other groups [14]. In our study, 78.9% of patients were Uygur, 15.8% were Kazakh, and no Han patients were observed, indicating a significantly higher prevalence of CF in the Uygur and Kazakh groups compared to the Han. The high prevalence of CF among Uygur and Kazakh populations likely stems from their mixed ancestry, shaped by interracial genetic admixture between East Asian and European lineages, with Caucasian ancestral components [8, 15]. Although CF was previously considered extremely rare in China, most reported cases have involved Han people, with limited data from Xinjiang [6, 7]. In this study, CF was diagnosed at a median age of 9.8 years, whereas symptom onset occurred at a median of 0.5 years and a diagnostic delay of approximately 6.3 years. This delay is likely attributable to limited clinical awareness of CF in Xinjiang, resulting in frequent misdiagnosis as recurrent pneumonia. Notably, three newly diagnosed children (Patients 16–18) from July 2025 received timely evaluations at ages 0.3–0.8 years, following regional initiatives to improve disease recognition. Furthermore, most patients (89.5%) were identified in the last four years, after January 2022, this is likely attributed to improvement in the diagnosis and increase awareness of the disease among doctors.

To date, more than 2,000 *CFTR* mutations have been identified worldwide, and the mutation spectrum has been well established in Caucasians. Previous studies have suggested a distinct *CFTR* mutation spectrum in Chinese, and there was minimal overlap between the variant spectra of Chinese and Caucasian CF patients [4–6]. For example, the most frequent mutation in Caucasians, p.F508del (about 78% allele frequency), has been observed only in six Chinese patients (1.8% allele frequency); while the variants c.2909G > A (p.G970D) and c.1766 + 5G > T are the most predominant variants in Chinese and show a significant Chinese ethnic tendency [4–6]. Notably, in this study, we identified 15 distinct mutations, with p.F508del being the most prevalent, accounting for 36.1% of *CFTR* alleles. We also observed mutations, c.1860T > G (p.H620Q), c.2991G > C (p.L997F), del ex4–11, c.3254 A > G (p.H1085R), c.2619 + 1G > A, c.349 C > T (p.R117C), c.3909 C > G (p.N1303K), and c.1911del (p.G637Hfs*26), previously unreported in Chinese populations. Conversely, the most frequent Chinese mutation (c.2909G > A, p.G970D) was detected in only Patient 11 (allele frequency of 2.8%). Thus, the findings of this study indicate that the spectrum of *CFTR* mutations in children with CF in Xinjiang differs from the previous reports in China, which mainly focused on the Han people. These observed differences can be attributed to Xinjiang’s unique geographical location and the prevalent Caucasian genetic background among the diverse ethnic composition, including the Uygur and Kazakh populations in the region. Importantly, given the higher prevalence of the F508del allele among children with CF in Xinjiang, *CFTR* modulator therapies are anticipated to emerge as a promising treatment option in this area. Moreover, recent clinical trials and real-world studies have demonstrated that the benefits of this targeted therapy could be even more pronounced when initiated earlier in life, specifically in younger children and infants [16]. Additionally, the proportion of homozygous mutations is higher than previously reported in China [6, 13].

A unique *CFTR* mutation spectrum may result in markedly different phenotypes in CF patients. In this study, the predominant clinical manifestations were respiratory diseases, such as chronic cough, recurrent pneumonia, sinusitis, bronchiectasis, and reduced pulmonary function, mirroring those reported in both Chinese and Caucasian CF populations. Consistent with previous reports in Chinese patients, the prevalence of ABPA (36.8%) was significantly higher than that reported in Caucasians (7–9%) [16]. This may be mainly attributed to the delayed diagnosis and longer disease course observed in our study. These factors are often associated with the predisposing factors of ABPA in CF, such as disease peaking in adolescence, the severity of lung disease, and colonization with *Pseudomonas* [17]. Given the high risk of ABPA, enhanced fungal surveillance and individualized immunomodulatory therapy should be considered in clinical practice. In this study, digestive system manifestations mainly include PI in 78.9% of patients and CFLD in 25.0%, both exceeding the rates reported in previous Chinese studies [13, 18–19]. This discrepancy may be attributed to the diverse profiles of *CFTR* gene mutations, as the digestive phenotype is consistently associated with severe *CFTR* genotypes, particularly p.F508del. Furthermore, given the high incidence of PI in Xinjiang, newborn screening (NBS) and pancreatic enzyme replacement therapy are strongly recommended. The FE assay has been proposed as a valuable NBS test for PI in CF patients, demonstrating good sensitivity and specificity [20–21]. Notably, in the current study, two newly diagnosed infant patients (Patients 16 and 18) exhibited low FE levels. This finding further suggests that FE can serve as a useful indicator for the early detection of CF among children in Xinjiang, particularly in settings where sweat testing is not readily available. Patient 1, with a homozygous mutation of (c.3196 C > T), ultimately died of cardiopulmonary failure, and patient 8, with compound heterozygous mutations (c.3196 C > T; c.4111_4113dup), exhibited the most severe destruction of lung structure. These findings imply that c.3196 C > T might be linked to more severe clinical manifestations.

The current study has certain limitations. Firstly, the sample size was small, and all patients were recruited from a single center, which may have introduced selection bias. Future studies with a larger sample size and multicenter collaborations across Xinjiang are warranted to validate the specificity of the phenotypic and genotypic spectra of CF in this region. Secondly, the majority of our patients did not undergo sweat testing due to the unavailability of this assay at our center, which is critical for confirming pathogenicity in newly identified mutations.

In conclusion, our study suggests that CF may be significantly underdiagnosed in Xinjiang, China. The genotypic spectrum of children with CF in Xinjiang is quite different from previous reports, which predominantly focused on the Han people. In this multiethnic region, p.F508del is the most frequent *CFTR* mutation. These findings offer fresh insights into CF in China, holding great significance for studying ethnicity-specific CF mutations and guiding future molecular genetic management in the Chinese CF population. Larger-scale studies are needed to confirm our findings and further delineate the phenotypic and genotypic spectra of CF in Xinjiang, China.

## Data Availability

The data that support the findings of this study are available from the corresponding author upon reasonable request.

## Abbreviations

ABPA: Allergic bronchopulmonary aspergillosis
BMI: Body mass index
CT: Computed tomography
CF: Cystic fibrosis
CFLD: Cystic fibrosis-related liver disease
CFTR: Cystic fibrosis trans-membrane conductance regulator gene
FE: Fecal elastase
FEV1: Forced expiratory volume in one second
IQR: Interquartile ranges
PI: Pancreatic insufficiency
*P. aeruginosa*: *Pseudomonas aeruginosa*
*S. aureus*: *Staphylococcus aureus*
Xinjiang: Xinjiang Uygur Autonomous Region
